# Toll-like receptor 4 modulates the cochlear immune response to acoustic injury

**DOI:** 10.1038/cddis.2016.156

**Published:** 2016-06-02

**Authors:** R R Vethanayagam, W Yang, Y Dong, B H Hu

**Affiliations:** 1 Center for Hearing and Deafness, State University of New York at Buffalo, 137 Cary Hall, 3435 Main Street, Buffalo, NY 14214, USA

## Abstract

Acoustic overstimulation traumatizes the cochlea, resulting in auditory dysfunction. As a consequence of acoustic injury, the immune system in the cochlea is activated, leading to the production of inflammatory mediators and the infiltration of immune cells. However, the molecular mechanisms responsible for initiating these immune responses remain unclear. Here, we investigate the functional role of Toll-like receptor 4 (Tlr4), a cellular receptor that activates the innate immune system, in the regulation of cochlear responses to acoustic overstimulation. Using a Tlr4 knockout mouse model, we examined how Tlr4 deficiency affects sensory cell pathogenesis, auditory dysfunction and cochlear immune activity. We demonstrate that Tlr4 knockout does not affect sensory cell viability under physiological conditions, but reduces the level of sensory cell damage and cochlear dysfunction after acoustic injury. Together, these findings suggest that Tlr4 promotes sensory cell degeneration and cochlear dysfunction after acoustic injury. Acoustic injury provokes a site-dependent inflammatory response in both the organ of Corti and the tissues of the lateral wall and basilar membrane. Tlr4 deficiency affects these inflammatory responses in a site-dependent manner. In the organ of Corti, loss of Tlr4 function suppresses the production of interleukin 6 (Il6), a pro-inflammatory molecule, after acoustic injury. By contrast, the production of inflammatory mediators, including Il6, persists in the lateral wall and basilar membrane. In addition to immune molecules, Tlr4 knockout inhibits the expression of major histocompatibility complex class II, an antigen-presenting molecule, in macrophages, suggesting that Tlr4 participates in the antigen-presenting function of macrophages after acoustic trauma. Together, these results suggest that Tlr4 regulates multiple aspects of the immune response in the cochlea and contributes to cochlear pathogenesis after acoustic injury.

Acoustic trauma, a leading cause of acquired sensory hearing loss in the adult population, is initiated by excessive mechanical stress to cochlear structures. This initial mechanical disturbance provokes multiple biological and molecular responses in the cochlea that control the final outcome of acoustic injury. As a defense mechanism, the cochlear immune system participates in the cochlear response to acoustic injury. In animal studies, the induction of the expression of immune mediators has been documented in the cochlea.^1, 2, 3^ These molecules include the pro-inflammatory mediators Tnf, Il6 and Il1b, which provoke an inflammatory response. Moreover, circulating monocytes infiltrate the cochlea^2, 4, 5, 6^ and transform into macrophages in a time- and site-dependent manner.^7^ Cochlear macrophages have been linked to the cochlear inflammatory response, dead cell clearance and antigen presentation.^5, 7, 8^ Although the inflammatory immune response has been implicated in cochlear pathogenesis and repair processes after acoustic injury, the molecular mechanisms responsible for initiating these immune responses remain unclear.

Toll-like receptor 4 (Tlr4) is a receptor for lipopolysaccharide, a structural component of the outer membrane of Gram-negative bacteria. This receptor also interacts with endogenous molecules of damaged tissues.^9, 10, 11, 12^ Upon binding its ligands, Tlr4 recruits adaptor molecules and activates multiple aspects of the inflammatory immune response, including the production of inflammatory molecules and the activation of immune cells via the NF-*κ*B signaling pathway.^13, 14, 15^ Tlr4 signaling has been implicated in many disease conditions, such as neurodegeneration, brain injury and ischemia.^16, 17^ In the cochlea, the role of Tlr4 in the pathogenesis of inner ear diseases is unclear. However, experimental evidence has implicated Tlr4-mediated signaling in the ototoxic effects of kanamycin and furosemide.^18^ In our recent study of transcriptomic changes in the cochlear sensory epithelium after acoustic injury,^19, 20^ we identified the Tlr4 signaling pathway as an upstream regulator of cochlear immune and inflammatory response. We found that Tlr4 was constitutively expressed in the cochlear sensory epithelium and that acoustic overstimulation caused the gene to be upregulated.^19^ Importantly, we found that the change in the expression of Tlr4 occurred in Deiters cells adjacent to the damaged sensory cells. These results suggest that Tlr4-mediated signaling is involved in the cochlear response to sensory cell damage.

The current study was designed to determine the role of Tlr4 in the pathogenesis of sensory cells and the modulation of the cochlear immune response after acoustic injury. We found that the lack of Tlr4 function decreases the magnitude of sensory cell damage and auditory dysfunction. We also found that a deficit in Tlr4 function alters the cochlear inflammatory response in a site-dependent manner. Moreover, we found that the loss of Tlr4 function suppresses the production of major histocompatibility complex class II (MHC II), an antigen presentation-related protein, in macrophages after acoustic injury. These observations suggest that Tlr4 contributes to multiple aspects of the cochlear immune response to acoustic stress and affects sensory cell survival after acoustic injury.

## Results

### Loss of Tlr4 function reduces the levels of sensory cell damage and cochlear dysfunction after acoustic injury

**Figure 1:**
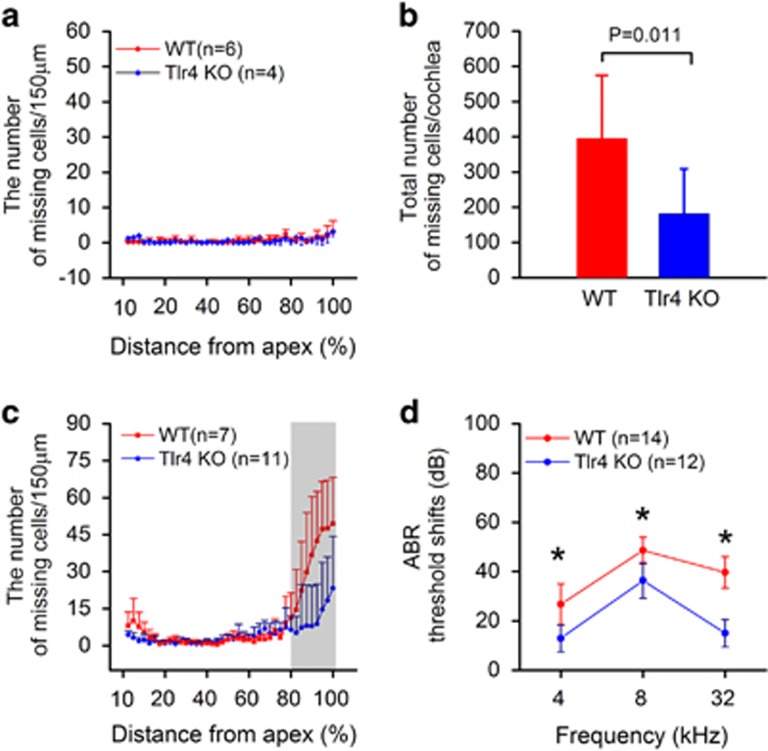
Loss of Tlr4 function reduces the level of sensory cell damage and cochlear dysfunction after acoustic injury. (**a**) Comparison of the average numbers of missing outer hair cells between Tlr4^−/−^ and wild-type mice under physiological conditions. Both strains display sporadic outer hair cell loss that was distributed along the length of the cochlea. There is no significant difference in the average numbers of missing cells between the two strains. (**b**) Comparison of the average numbers of missing outer hair cells per cochlea between Tlr4-deficient and wild-type mice 20 days after acoustic trauma. The average number of missing outer hair cells is significantly greater in the wild-type mice than in Tlr4 knockout mice (Student's *t* test, t (16)=2.89, *P*=0.011). (**c**) Cochleogram showing a comparison of the distribution of missing outer hair cells along the cochlea between Tlr4-deficient and wild-type mice 20 days after acoustic trauma. The shaded area illustrates a significant reduction in the number of missing outer hair cells at the basal end of the cochlea in the Tlr4 knockout mice. (**d**) Comparison of ABR threshold shifts between the Tlr4^−/−^ mice and the wild-type mice measured at 20 days after acoustic trauma. The threshold shifts in the knockout mice are significantly less pronounced than those observed in wild-type mice (two-way ANOVA, *F* (1, 78)=144.8; *P*<0.001). Asterisks indicate *P*<0.05 (Tukey test). *n*=the number of cochleae

We previously demonstrated that Tlr4 is constitutively expressed in the organ of Corti and that its expression is upregulated after acoustic overstimulation.^19^ Here, we sought to determine whether Tlr4 contributes to sensory cell pathogenesis after acoustic injury. Under physiological conditions, the sporadic loss of outer hair cells was observed. This spontaneous cell death is likely associated with the Cdh23^ahl^ mutation, which causes age-related hearing loss in these mice.^21^ As shown by the cochleogram (Figure 1a), both Tlr4 knockout mice (*n*=4 cochleae) and wild-type mice (*n*=6 cochleae) displayed spontaneous sensory cell loss, and the average numbers of missing cells per cochlea were similar (23.3±9.0 for wild-type cochleae *versus* 28.3±14.7 for Tlr4-deficient cochleae; Student's *t* test, t (8)=0.66, *P*=0.52). This observation suggests that Tlr4 deficiency does not affect the viability of sensory cells under physiological conditions.

We then examined the cochlear pathology at 20 days after acoustic injury. In wild-type mice (*n*=7 cochleae), the average number of missing cells per cochlea was 394±181. By contrast, the average number was 181±131 in Tlr4 knockout mice (*n*=11 cochleae), which is significantly less than that observed in the wild-type mice (Figure 1b, Student's *t* test, t (16)=2.89, *P*=0.011). Notably, this reduction occurred primarily at the basal end of the cochlea, where hair cell lesions were present (Figure 1c). Consistent with this pathological finding, the measurement of ABR thresholds before and 20 days after acoustic injury revealed a reduced level of threshold shifts in the Tlr4 knockout mice (Figure 1d; two-way ANOVA, *F* (1, 78)=144.8; *P*<0.001; Tukey pairwise comparison, *P*<0.05 for 4, 8 and 32 kHz). These findings indicate that Tlr4 deficiency reduces the level of sensory cell damage and auditory dysfunction after acoustic injury.

### Depletion of Tlr4 affects the expression of inflammatory molecules in the organ of Corti after acoustic injury

**Figure 2:**
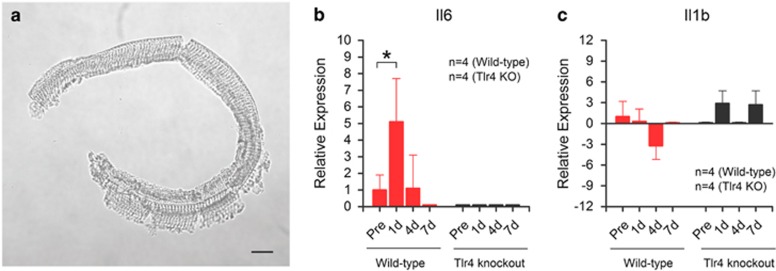
Comparison of the transcriptional expression of inflammatory genes in the organs of Corti of Tlr4 knockout mice and wild-type mice. Five pro-inflammatory genes (Il6, Il1b, Tnf, Ccl2 and Ccl4) were examined in organs of Corti collected before and at three time points (1, 4 and 7 days) after acoustic injury. Among the five examined genes, two (Il6 and Il1b) were detected in the organ of Corti; these data are presented in the figure. (**a**) A micrograph showing a typical organ of Corti used for the expression analysis of pro-inflammatory genes. Bar=60 *μ*m. (**b**) A comparison of Il6 expression between Tlr4 knockout and wild-type mice. The expression level is significantly increased at 1 day after acoustic injury (one-way ANOVA, *F* (2, 9)=5.79, *P*=0.024; Tukey test, *P*=0.036). By 4 days after injury, the increase level has decreased to the pre-noise level (Tukey test, *P*=0.99). At the 7-day time point, expression was not detected. For Tlr4 knockout mice, the expression of Il6 was detected neither before nor after acoustic injury. (**c**) Comparison of Il1b expression between wild-type and Tlr4 knockout mice. Although the expression levels vary among groups, none of these differences are statistically significant

The finding that sensory cell damage was reduced in Tlr4^−/−^ mice prompted us to assess whether Tlr4 deficiency affects the inflammatory response of the organ of Corti to acoustic injury. The organ of Corti was chosen for study because this tissue contains sensory cells and their neighboring supporting cells (Figure 2a).

We examined the mRNA expression of five pro-inflammatory genes (Il6, Il1b, Tnf, Ccl2 and Ccl4) that are known to respond to cochlear stresses, including acoustic injury.^1, 22^ The transcriptional expression of these genes was examined before as well as 1, 4 and 7 days after acoustic trauma (*n*=4 biological replicates collected from four individual animals for each time point). These time points were selected because they span the major period of cochlear pathogenesis.^23, 24^ Using a raw qRT-PCR Ct value of 35 as the cutoff value, we determined which of these genes were expressed in the organ of Corti. Among the five examined genes, three (Tnf, Ccl2 and Ccl4) were detected in neither wild-type nor Tlr4^−/−^ mice. The two remaining genes (Il6 and Il1b) were detected, and their expression levels were strain-dependent. In wild-type mice, Il6 was detected under normal conditions, and its expression was significantly upregulated 1 day post noise exposure (one-way ANOVA, *F* (2, 9)=5.79, *P*=0.024; Tukey test, *P*=0.036; Figure 2b). This finding is consistent with the result of a previous investigation showing an early upregulation of Il6 after acoustic injury.^1^ In Tlr4 knockout mice, Il6 expression was not detected under physiological conditions, and its expression remained undetectable after acoustic injury. This observation suggests that Il6 response to acoustic injury is Tlr4-dependent. Because Il6 is an important pro-inflammatory mediator in the cochlea,^1, 2^ our finding suggests that Tlr4 affects the inflammatory response of the organ of Corti to acoustic injury.

Il1b was detected in wild-type mice under physiological conditions. Its expression was downregulated at 4 days after noise exposure; however, this change was not statistically significant (one-way ANOVA, *P*>0.05). The expression of Il1b was not detected in Tlr4 KO mice under physiological conditions. After noise exposure, its expression was upregulated at the 1- and 7-day time points. Again, these changes were not statistically significant (one-way ANOVA, *P*>0.05) because of large individual variation.

### Expression of Tlr4 in non-organ of Corti tissues of the cochlea

**Figure 3:**
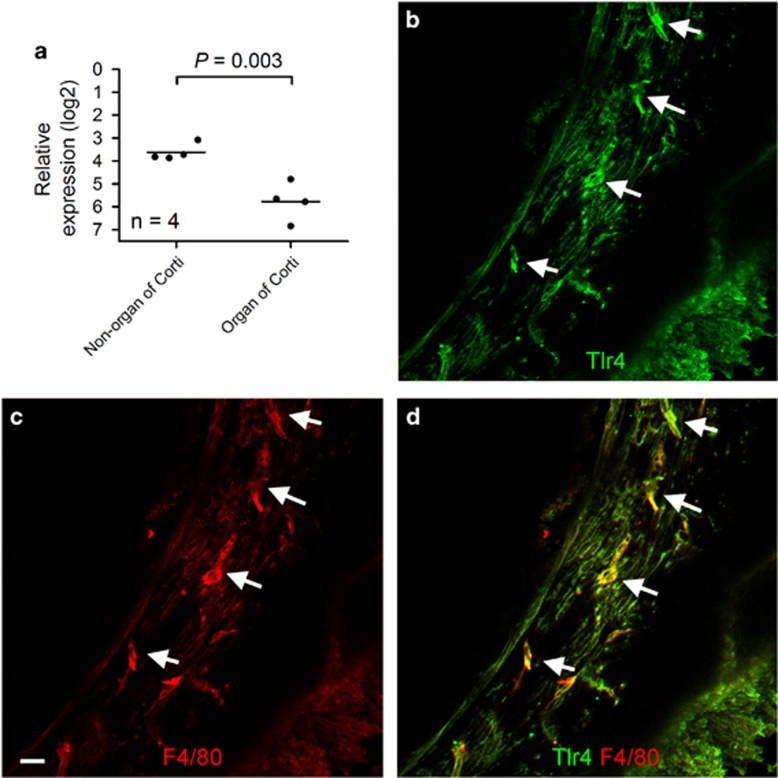
Tlr4 expression in non-organ of Corti tissues. (**a**) Constitutive expression of Tlr4 mRNA in the organ of Corti and non-organ of Corti tissues. The non-organ of Corti tissues contain the lateral wall and the basilar membrane, where immune cells reside. The dots represent individual values, and the horizontal lines represent the group mean. The expression levels are normalized using two reference genes (Rpl13a and Hsp90ab1). The average expression level of Tlr4 in the non-organ of Corti tissues was significantly higher than in the organ of Corti (Student's *t* test, t (6)=4.72, *P*=0.003). (**b**) A typical image showing Tlr4 immunoreactivity in cells beneath the basilar membrane. Arrows indicate the positive cells with strong Tlr4 immunoreactivity. (**c**) Double-labeling of F4/80, a macrophage marker protein, in the same tissue. Bar=20 *μ*m. (**d**) Merged image of (**b** and **c**). Notice that the cells with strong Tlr4 immunoreactivity are also F4/80-positive

The lateral wall and the basilar membrane, which we collectively refer to as the non-organ of Corti tissues, have an important role in the maintenance of cochlear homeostasis. We sought to determine whether Tlr4 regulates the inflammatory response to acoustic injury in these tissues. We first examined whether Tlr4 is expressed in the lateral wall and the basilar membrane. Using qRT-PCR, we observed strong constitutive expression of Tlr4 (Figure 3a) in wild-type mice (*n*=4 cochleae) and no expression in Tlr4 knockout mice (data not shown), as expected. For comparative purposes, we also examined the expression level of Tlr4 in the organ of Corti. Tlr4 expression was detected in wild-type mice (*n*=4 cochleae), but was not detected in Tlr4 knockout mice (*n*=4 cochleae), which is consistent with our previously reported observation.^19^ Notably, Tlr4 expression in the non-organ of Corti tissues was significantly higher than in the organ of Corti (Student's *t* test, t (6)=4.72, *P*=0.003).

We also examined Tlr4 protein expression using immunohistochemistry. We observed Tlr4-positive cells on the scala tympani side of the basilar membrane (Figure 3b). These Tlr4-positive cells displayed strong F4/80 immunoreactivity, a macrophage marker, indicating that these cells were macrophages (Figures 3c and d). In addition, Tlr4 immunoreactivity was found in the mesothelial cells of the basilar membrane. However, the staining intensity of these cells was considerably weaker than that of the macrophages. Together, these observations indicate that Tlr4 is expressed in the macrophages of the non-organ of Corti tissues.

### Continued production of pro-inflammatory molecules in the non-organ of Corti tissues of Tlr4-deficient mice

**Figure 4:**
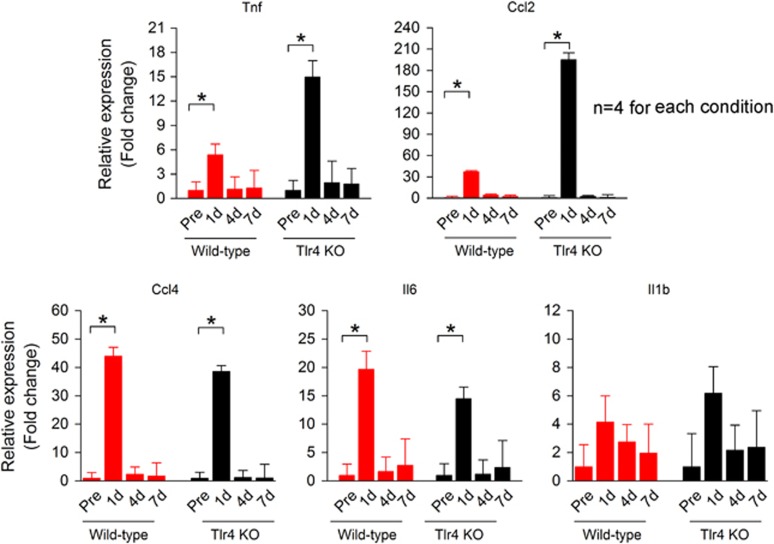
The transcriptional expression of pro-inflammatory genes in non-organ of Corti tissues after acoustic injury. The expression of five pro-inflammatory genes (Tnf, Ccl2, Ccl4, Il6 and Il1b) was examined in cochlear samples containing the lateral wall and the basilar membrane tissues before and at three time points (1, 4 and 7 days) after acoustic injury. In wild-type mice (red columns), all examined genes exhibited time-dependent upregulation after acoustic injury. Four genes (Tnf, Ccl2, Ccl4 and Il6) exhibited significant upregulation at 1 day post noise exposure (one-way ANOVA with Tukey test for pairwise comparison, *P*<0.05). Il1b expression is also increased at this time point, but the difference is not statistically significant. At 4 and 7 days after acoustic injury, the expression levels of the examined genes return to the pre-noise levels. Black columns illustrate the expression changes observed in Tlr4^−/−^ mice. These mice display a similar pattern of expression, with a significant upregulation at 1 day after acoustic injury for all examined genes except for Il1b (one-way ANOVA with Tukey test for pairwise comparison, *P*<0.05). Again, the expression levels at 4 and 7 days after acoustic injury return to or close to the pre-noise level. These observations suggest that loss of Tlr4 does not attenuate the inflammatory response in non-organ of Corti tissues

We wanted to determine whether the loss of Tlr4 function affects the inflammatory response in non-organ of Corti tissues. We therefore examined the transcriptional expression of five pro-inflammatory genes (Tnf, Ccl2, Ccl4, Il6 and Il1b) before and at three time points (1, 4, and 7 days) after acoustic injury (*n*=4 biological replicates for each condition). All examined molecules were detected under physiological conditions. This discovery rate (five out of five examined genes) is markedly greater than that observed in the organ of Corti (two out of five examined genes), suggesting greater immune activity in the non-organ of Corti tissues. The expression of these genes displayed a time-dependent upregulation after acoustic trauma. In wild-type mice, four genes (Tnf, Ccl2, Ccl4 and Il6) were significantly upregulated at 1 day after acoustic injury (one-way ANOVA, *F* (3, 12)=7.23, 5.96, 31.62 and 16.57; *P*=0.007, 0.01, 0.001 and 0.001 for Tnf, Il6, Ccl2 and Ccl4, respectively; Tukey test, *P*<=0.01; Figure 4). This elevated expression returned to pre-noise levels by 4 days after acoustic injury. This observation suggests that acoustic injury provokes an inflammatory response during the early phase of cochlear pathogenesis. Like wild-type mice, Tlr4 knockout mice exhibited a time-dependent upregulation of the pro-inflammatory genes. All examined genes except for Il1b displayed a significant upregulation at 1 day after acoustic injury (one-way ANOVA, *F* (3, 12)=8.87, 4.10, 19.44 and 23.21; *P*=0.002, 0.032, 0.001 and 0.001 for Tnf, Il6, Ccl2 and Ccl4, respectively; Tukey test *P*<0.05 for all of the genes; Figure 4). These increased levels returned to or close to pre-noise levels by 4 days after acoustic injury. This observation suggests that the lack of Tlr4 function does not abolish the pro-inflammatory response in the non-organ of Corti tissues.

### Lack of Tlr4 does not prevent the infiltration of monocytes into the cochlea, nor the transformation of monocytes into macrophages after acoustic trauma

**Figure 5:**
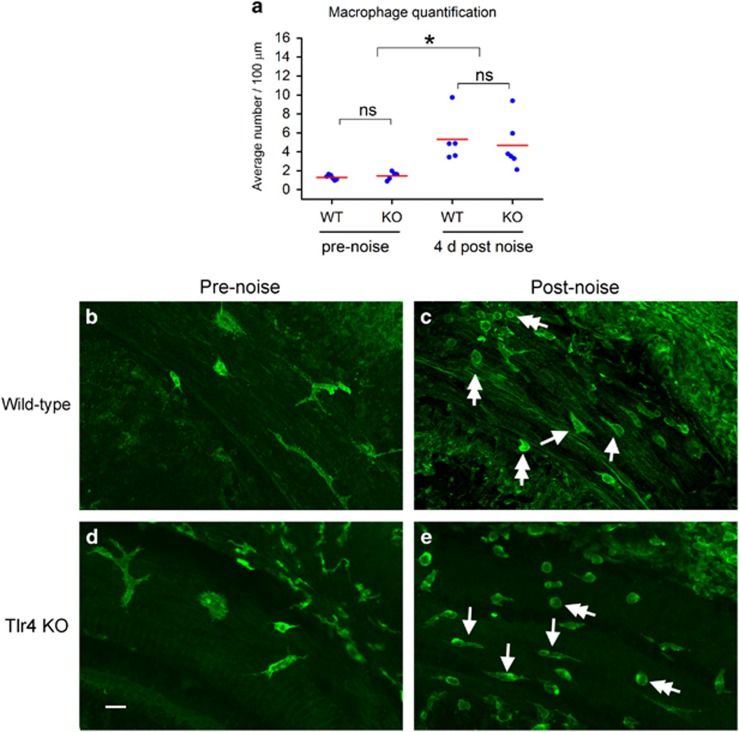
Comparisons of noise-induced monocyte infiltration and transformation between wild-type and Tlr4 knockout mice. Monocytes and macrophages were identified by immunolabeling CD45, a marker of bone marrow–derived immune cells. (**a**) The average number of CD45-positive cells in wild-type and the Tlr4^−/−^ mice before and 4 days after acoustic injury. Before noise exposure, tissue macrophages are present in both wild-type and Tlr4^−/−^ mice. There is no significant difference in the number of macrophages between these two strains of mice (two-way ANOVA, *P*>0.05). Four days after acoustic injury, the numbers of cells are significantly increased in both strains (two-way ANOVA, Tukey pairwise comparison, *P*<0.05). However, there is no significant difference between the two types of mice (*P*>0.05). (**b** and **c**) Representative images showing the morphology of monocytes and macrophages in wild-type cochleae examined before (**b**) and 4 days after acoustic injury (**c**). Double arrows point to the round CD45-positive cells, which exhibit monocyte morphology. Arrows point to the CD45-positive cells showing an elongated cell body, a phenotype typical of monocyte transformation to macrophage. (**d** and **e**) Typical images showing the morphology of monocytes and macrophages in Tlr4-null cochleae examined before (**d**) and 4 days after acoustic injury (**e**). Double arrows point to the cells with monocyte morphology, and arrows indicate the cells with a transforming monocyte phenotype. Bar=20 *μ*m

Acoustic trauma is known to induce a time-dependent infiltration of monocytes into the cochlea.^2, 4, 5, 6^ Our recent study further documented the transformation of the infiltrated monocytes into macrophages.^7^ We therefore wanted to determine whether Tlr4 deficiency affects these immune cell activities. We first quantified the number of macrophages beneath the basilar membrane. Under steady-state conditions, the wild-type (*n*=6 cochleae) and Tlr4 knockout mice (*n*=5 cochleae) exhibited a similar level of CD45-positive cells (Student's *t* test, t (9)=−0.788, *P*=0.451; Figure 5a), suggesting that Tlr4 deficiency does not affect tissue macrophages in the cochlea. Four days after acoustic injury, the wild-type mice (*n*=5 cochleae) displayed a marked increase in the number of CD45-positive cells, which is consistent with our previous observations.^7^ The knockout mice (*n*=6 cochleae) also displayed a marked increase in the number of CD45-positive cells at a level similar to that of wild-type mice (Mann–Whitney Rank Sum Test, t=34.0, *P*=0.523; Figure 5a). This analysis suggests that Tlr4 deficiency does not prevent the infiltration of monocytes into the cochlea after acoustic injury.

We then determined whether the loss of Tlr4 function affects the transformation of monocytes into macrophages. In wild-type mice, macrophages were irregularly shaped, with long-thick or short-fine projections under steady-state conditions (Figure 5b). After acoustic injury, monocytes infiltrated the cochlea. These cells had a small round shape (Figure 5c). Many cells displayed short projections, a phenotype representing the transformation of monocytes.^7^ Like wild-type mice, the Tlr4^−/−^ mice exhibited irregularly shaped macrophages under steady-state conditions (Figure 5d). After acoustic injury, small round CD45-positive cells (i.e., monocytes) were observed (Figure 5e). Moreover, many cells exhibited elongated cell bodies with thin projections, suggesting the transformation from monocytes into macrophages. These results suggest that Tlr4 deficiency does not prevent the infiltration of monocytes into the cochlea or the transformation of monocytes into macrophages.

### Tlr4 deficiency suppresses the induction of MHC II expression after acoustic trauma

**Figure 6:**
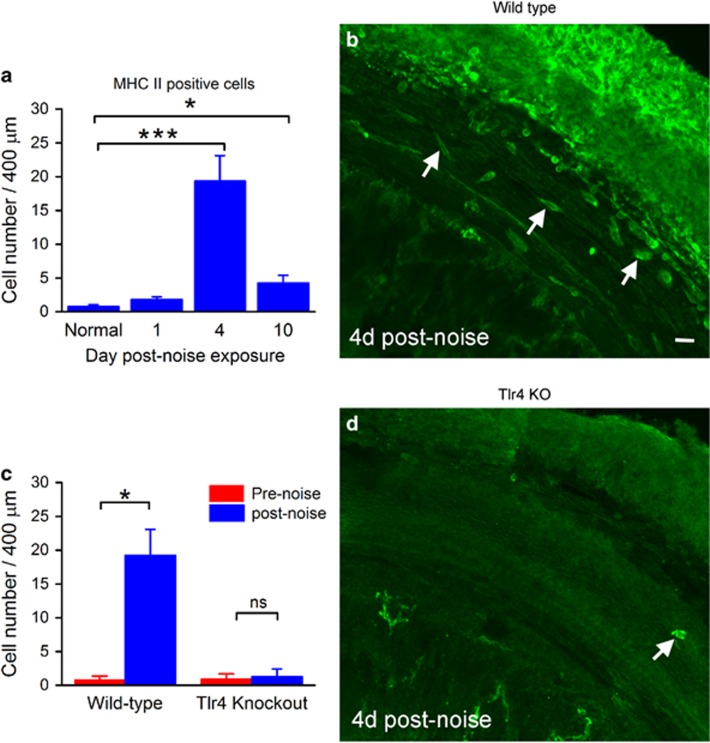
Comparisons of the noise-induced expression of MHC II between wild-type and Tlr4 knockout mice. (**a**) Quantification of MHC II-positive cells identified beneath the basilar membrane before and at three time points (1, 4 and 10 days) after noise exposure in wild-type mice (*n*=5 cochleae). The number is significantly increased at 4 days after noise exposure (one-way ANOVA, *P*<0.001; Tukey test, *P*<0.001 indicated by the three asterisks). While this increase is reduced at 10 days after noise exposure, the number of positive cells remains significantly higher than that observed in the control ears (Tukey test, *P*=0.045 indicated by the asterisk). (**b**) A typical image showing the immunolabeling of MHC II in a wild-type mouse examined 4 days after acoustic injury. Arrows point to the MHC II-positive cells beneath the basilar membrane. Bar=20 *μ*m. (**c**) Comparison of the noise-induced increase in the numbers of MHC II-positive cells in wild-type and Tlr4^−/−^ mice. There is a significant increase in the number of MHC II-positive cells in wild-type mice (two-way ANOVA, Tukey test, *P*<0.05). By contrast, the number remained unchanged in Tlr4-deficient mice after acoustic injury (two-way ANOVA, Tukey test, *P*>0.05). (**d**) A typical image showing MHC II immunolabeling in a Tlr4-deficient mouse examined 4 days after acoustic injury. The arrow points to an MHC II-positive cell. Bar=20 *μ*m

**Figure 7:**
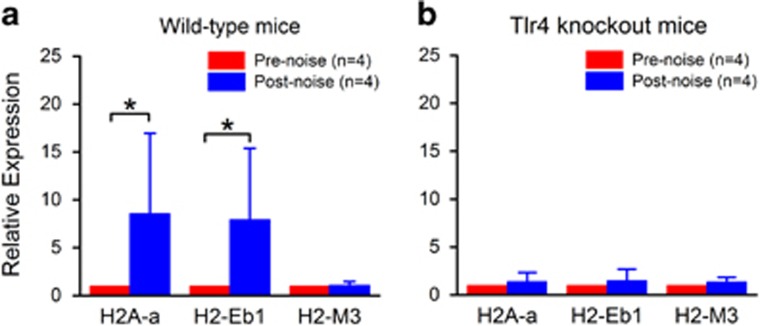
Comparison of transcriptional expression of MHC II-encoding genes between wild-type and Tlr4^−/−^ mice. (**a**) Comparison of the expression levels of H2A-a, H2-Eb1 and H2-M3 before and 4 days after acoustic injury in wild-type mice. There is a significant increase in the expression levels of H2A-a and H2-Eb1 after acoustic injury (Student's *t* test; *P*=0.015 for H2-Aa; *P*=0.017 for H2-Eb1). (**b**) Comparison of the expression levels of the three genes before and 4 days after acoustic injury in Tlr4^−/−^ mice. There is no significant increase in the expression levels of all examined genes after acoustic injury (Student's *t* test, *P*>0.05)

Antigen presentation is an important immune function that links the innate immune response to the adaptive immune response. This function is activated in cochlear macrophages after acoustic injury.^7^ We wanted to determine whether Tlr4 deficiency affects this immune function. First, we examined the macrophage expression of MHC II, an antigen-presenting protein. In wild-type mice (*n*=5 cochleae), MHC II-positive cells were located primarily in the apical section of the cochlea, in numbers ranging from 4 to 9 per cochlea under steady-state conditions. After acoustic injury, there was a time-dependent increase in the number of the MHC II-positive cells (*n*=4 cochleae for each time point; one-way ANOVA, *F* (3, 14)=88.97, *P*<0.001; Figure 6a) with a significant increase at 4 days after acoustic injury (Figure 6b, Tukey test, *P*<0.001). Like wild-type mice, the Tlr4 knockout mice exhibited sporadic MHC II-positive cells totaling 4–10 per cochlea under steady-state conditions (*n*=11 cochleae). However, unlike the wild-type cochleae, Tlr4^−/−^ mice (*n*=4 cochleae) showed no significant increase in the number of MHC II-positive cells 4 days after acoustic injury (Figures 6c and d; two-way ANOVA, strain–noise interaction, *F*=151.522, *P<*0.001; Tukey test, *P*>0.05). We also examined the transcriptional expression of three members of the MHC II gene family (H2-Aa, H2-Eb1 and H2-M3) that encode the MHC II chain.^25^ In the wild-type mice, H2-Aa and H2-Eb1 were significantly upregulated at 4 days after acoustic injury (Student's *t* test; t (6)=3.63, *P*=0.015 for H2-Aa; t (6)=3.53, *P*=0.017 for H2-Eb1, Figure 7a). In the Tlr4^−/−^ mice, there was no significant change in the expression of these genes (Figure 7b, Student's *t* test, *P*>0.05). Together, these observations suggest that Tlr4 is required for the production of MHC II in macrophages after acoustic injury.

**Figure 8:**
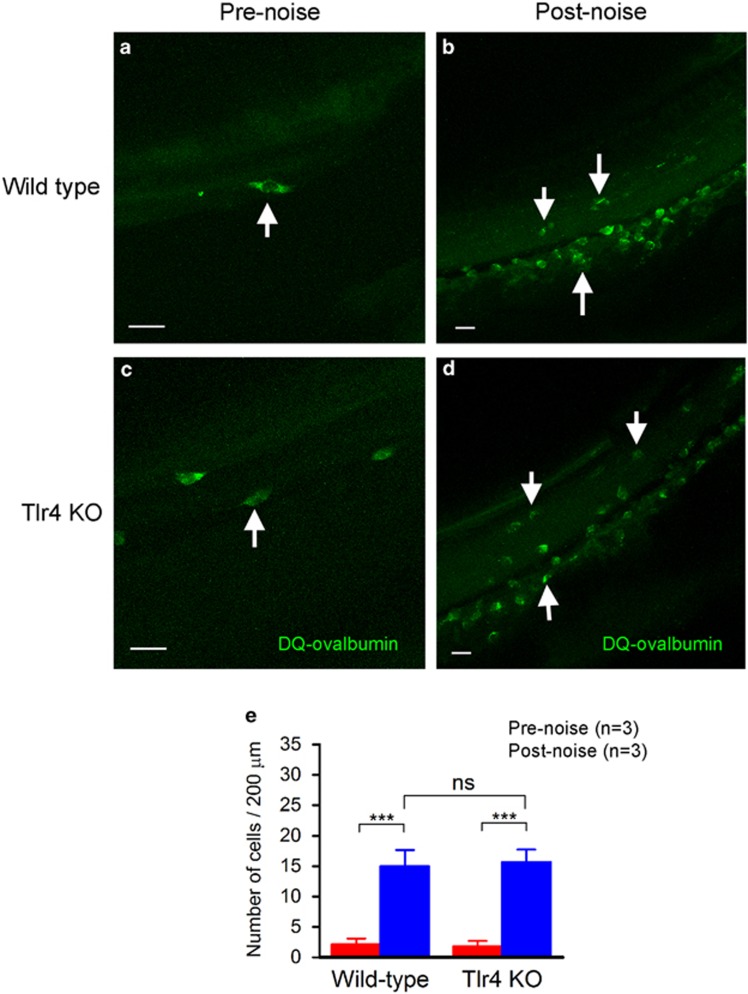
Comparison of DQ-Ovalbumin-positive cells between wild-type and Tlr4 knockout mice. The cochleae were incubated with DQ-Ovalbumin, which is a self-quenching conjugate of albumin exhibiting bright green fluorescence upon endo-lysosomal degradation. (**a** and **b**) DQ-Ovalbumin staining in wild-type mice under normal conditions (**a**) and 4 days after acoustic injury (**b**). Arrows point to positively stained cells. Note that the number of positive cells in the noise-damaged cochlea is markedly greater than in the normal cochlea. (**c** and **d**) DQ-Ovalbumin staining in Tlr4-deficient mice before (**c**) and 4 days after acoustic injury (**d**). Like wild-type mice, knockout mice exhibit an increase in the uptake of DQ-Ovalbumin by macrophages. (**e**) Quantification of DQ-Ovalbumin-positive cells in wild-type and Tlr4 knockout mice. Both wild-type mice and Tlr4-deficient mice display an increase in the number of positive cells (two-way ANOVA, *F*=191.4 *P*<0.001). There is no significant difference in the level of the increase between the two species (*F*=0.028, *P*>0.05). Bars=20 *μ*m

Antigen presentation requires cellular processing of antigens. We wanted to determine whether Tlr4 is required for these antigen-processing functions in macrophages. To this end, we examined the ability of macrophages to process ovalbumin that had been conjugated with a fluorescent probe. Under physiological conditions, less than 10 positive cells were observed beneath the basilar membrane in wild-type mice (*n*=3 cochleae, Figure 8a). In cochleae examined 4 days after noise exposure (*n*=3 cochleae), the number of positive cells was significantly increased (Figure 8b). Like wild-type mice, Tlr4-deficient mice exhibited a few positive cells beneath the basilar membrane under normal conditions (*n*=3 cochleae, Figure 8c). The number of positive cells was also increased 4 days after acoustic injury (Figure 8d, *n*=3 cochleae). There was no significant difference between the wild-type and the knockout mice (two-way ANOVA, *F*=0.028, *P*>0.05; Figure 8e), suggesting that Tlr4 knockout does not affect the phagocytic and proteolytic functions of macrophages within the cochlea.

## Discussion

Sensory cell degeneration is a major consequence of acoustic injury. This degenerative process activates the immune defense response of the cochlea. Our previous study has revealed the induction of Tlr4 expression in the supporting cells neighboring damaged sensory cells,^19^ suggesting that sensory cell damage activates the Tlr4 response. Here, we present the novel finding that a deficit in Tlr4 signaling reduces the level of sensory cell damage. This observation suggests that Tlr4 activation in the organ of Corti exerts a detrimental effect on sensory cells after acoustic injury. Considering the known pro-inflammatory role of Tlr4 signaling in the anti-microbial activity of non-cochlear tissues,^26, 27^ we suspect that the effect of Tlr4 on cochlear pathogenesis is mediated by its role in the induction of pro-inflammatory molecules. This view is supported by our expression analysis of pro-inflammatory mediators in Tlr4-deficient and wild-type mice. Specifically, the loss of Tlr4 function prevents the noise-induced expression of Il6, an important pro-inflammatory mediator in the cochlea.^1, 2, 3^ These observations suggest that Tlr4 promotes noise-induced inflammation in the organ of Corti. Unlike in the organ of Corti, the induction of the expression of pro-inflammatory mediators persists in the lateral wall and the basilar membrane tissues in Tlr4 knockout mice after acoustic injury. This result suggests that Tlr4 deficiency is not sufficient to suppress the induction of the inflammatory response in these non-organ of Corti tissues. This finding is not surprising, given that the cochlea exhibits constitutive expression of other TLR family members and that their expression is also upregulated after acoustic injury.^19^ These molecules could have a role in the activation of the inflammatory response in the absence of Tlr4 function.

The lateral wall and the basilar membrane contain immunocompetent cells, including mononuclear phagocytes and fibrocytes.^28, 29, 30, 31, 32, 33^ These cells have been linked to the production of pro-inflammatory mediators under stress, but their contributions to the cochlear inflammatory response are unclear. Here, we observed a discrepancy in the timing of monocyte infiltration and immune molecule production. We and others have reported that the peak level of monocyte infiltration and transformation appears to occur between 3 and 7 days after acoustic trauma.^2, 4, 5, 6, 7^ However, the peak level of the production of pro-inflammatory molecules occurs 1 day after acoustic injury. This discrepancy suggests that the infiltrated monocytes are not a major contributor to the early production of inflammatory mediators. Instead, cochlear resident cells, such as fibrocytes and tissue macrophages that reside in the cochlea under steady-state condition, are likely to contribute to the early production of inflammatory molecules in the lateral wall and the basilar membrane.

Antigen presentation is an important immune function that bridges the innate and the adaptive immune responses. We previously demonstrated the activation of the antigen-presenting function in cochlear macrophages after acoustic injury,^7^ which is consistent with a previous study of the induction of MHC II expression following immune challenge by sterile labyrinthitis in the cochlea.^34^ Here, we demonstrated that the lack of Tlr4 function suppresses the noise-induced production of MHC II, suggesting that Tlr4 participates in noise-induced antigen-presentation activity. Notably, Tlr4 deficiency does not affect the internalization and proteolysis of antigen molecules by macrophages, as evidenced by the continued uptake and cellular processing of DQ-Ovalbumin. This observation suggests that the phagocytic and proteolytic functions of macrophages are preserved in Tlr4^−/−^ mice. The molecular mechanism underlying Tlr4-dependent control of MHC II antigen presentation is not completely clear. Toll-like receptor signaling has been found to control multiple steps of antigen handling and presentation.^35, 36^ Our current study suggests a role for Tlr4 in presenting processed antigens by cochlear macrophages. Because antigen presentation is an important step for the activation of the adaptive immune response and because the activation of adaptive immune response can exert long-term effects on cochlear homeostasis, future investigations of the role for Tlr4 in signal transition from the innate response to the adaptive response are expected to shed light on the long-term effect of acoustic injury on cochlear homeostasis.

The cochlea has multiple partitions; each consists of distinct cell populations and has unique functional roles in cochlear homeostasis. The difference in the immune capacity of the organ of Corti and its surrounding non-organ of Corti tissues is not completely clear. Here, we provide evidence that the lateral wall and basilar membrane tissues exhibit a greater immune capacity than the organ of Corti. The organ of Corti lacks professional immune cells,^7, 37^ whereas the lateral wall and the basilar membrane are rich in professional and non-professional immune cells.^28, 29, 30, 31, 32, 33^ This difference could contribute to the difference in the production of immune mediators between the two tissue types. Although the organ of Corti lacks immune cells, it does have an immune capacity, which has been linked to its resident cells, particularly supporting cells.^19^ Our current finding that Tlr4 deficiency affects both sensory cell pathogenesis and the production of inflammatory mediators in the organ of Corti suggests that the immune activity in this tissue can exert a direct impact on sensory cell pathogenesis after acoustic injury. Whereas the precise contribution of inflammatory mediators in non-organ of Corti tissues to sensory cell pathogenesis is unknown, the strong activity of inflammatory mediators is likely to attract immune cells to these regions, which could affect multiple functions, such as the removal of damaged cells, promotion of inflammation and facilitation of the cochlear recovery process.

## Materials and Methods

### Animals

C57BL/6 J and B6.B10ScN-Tlr4^lps-del^/JthJ mice (4–6 weeks, equal numbers of male and female, The Jackson Laboratory, Bar Harbor, ME, USA) were used. The animals were housed at 22±1 °C under a standard 12-h light/dark cycle and were allowed free access to water and a regular mouse diet (the standard Harlan 2018). To reduce the usage of animals while maintaining sufficient sample sizes, both cochleae of each animal were collected and assigned to different assays. The number of cochleae used for each experiment is described in the Results section and in the figures. All procedures involving the use and care of the animals were approved by the Institutional Animal Care and Use Committee of the State University of New York at Buffalo.

### Noise exposure

Acoustic injury was induced by exposure to continuous noise (1–7 kHz) at 120 dB (sound pressure level, re 20 *μ*Pa) for 1 h. This level of noise exposure was used because it causes permanent hearing loss and sensory cell death,^19^ allowing us to determine the cochlear response to sensory cell damage. The mice were individually exposed to the noise in a holding cage. The noise signal was generated using a real-time signal processor (RP2.1, Tucker Davis Technologies, TDT, Alachua, FL, USA). The signal was routed through an attenuator (PA5 TDT) and a power amplifier (Crown XLS 202, Harman International Company, Elkhart, IN, USA) to a loudspeaker (NSD2005-8, Eminence, Eminence, KY, USA). The speaker was positioned 30 cm above the animal. The noise level was calibrated using a sound level meter (LD-PCB, model 800 B, APCB Piezotronics Div., Larson Davis, Depew, NY, USA), a pre-amplifier (LD-PCB, model 825, Larson Davis) and a condenser microphone (Larson and Davis, LDL 2559).

### Auditory brainstem responses (ABR)

ABR thresholds were measured to assess the auditory function of the mice as previously described.^38^ The mice were anesthetized with an intraperitoneal injection of a mixture of ketamine (87 mg/kg) and xylazine (3 mg/kg). The body temperature was maintained at 37.5 °C with a warming blanket (Homeothermic Blanket Control Unit, Harvard Apparatus, Holliston, MA, USA). Stainless-steel needle electrodes were placed subdermally over the vertex (noninverting input) and posterior to the stimulated and unstimulated ears (inverting input and ground) of the animal. The ABRs were provoked with tone bursts at 4, 8 and 32 kHz (0.5 ms rise/fall Blackman ramp, 1 ms duration, alternating phase) using a presentation rate of 21/s. The tone bursts were generated digitally (SigGen, Alachua, FL, USA) using a D/A converter (RP2.1; TDT; 100 kHz sampling rate) and were fed to a programmable attenuator (PA5; TDT), an amplifier (SA1; TDT) and a magnetic speaker (MF1 multi-field magnetic speaker; TDT). The electrode outputs were collected using a pre-amplifier/base station (RA4LI and RA4PA/RA16B; TDT). The responses were filtered (100–3000 Hz), amplified and averaged using TDT hardware and software. The ABR threshold was defined as the lowest intensity that reliably elicited a detectable response. Using the pre-noise thresholds as the baseline, the threshold shifts induced by acoustic overstimulation were calculated. The average ABR threshold shifts between the wild-type and Tlr4 knockout mice were compared using two-way ANOVA with the two factors strain × frequency. If significant main effects were identified, the Tukey *post hoc* test was used to evaluate the interaction between the factors.

### Assessment of sensory cell damage

To determine the magnitude of sensory cell damage, we quantified the number of missing outer hair cells along the organ of Corti from the apex to the base of the cochlea at 20 days after acoustic trauma when the cochlear pathology had stabilized. The animals were killed by CO_2_ asphyxiation and were decapitated. The cochleae were collected and then fixed with 10% buffered formalin overnight at 4 °C.

Cochlear dissection for sensory cell inspection in the mouse presents a technical challenge. To prevent dissection damage, we developed a method of *in situ* observation of the cochlear sensory epithelium. Specifically, the dissection and observation were performed in two steps. First, the bony shell over the apex of the cochlea was opened and the lateral wall was trimmed slightly. The entire cochlea was stained with Alexa Fluor 488 phalloidin (1 : 75, Applied Biosystems, Carlsbad, CA, USA) in 10 mM phosphate-buffered saline (PBS) at room temperature in the dark for 30 min. After staining, the cochlea was placed in a culture dish containing distilled water. The apex of the cochlea was inspected, and the sensory cells were photographed under a fluorescence microscope (Leica Z6 APO Manual MacroFluo, × 10 objective) equipped with a Leica DFC digital camera. After imaging, the cochlea was further dissected to remove the bony shell covering the middle and basal portions. Then, the phalloidin staining was repeated, and the organ of Corti was photographed using a method identical to that used for the inspection of the apical section of the cochlea. Each organ of Corti was thoroughly examined from the apex to the base of the cochlea. The number of missing outer hair cells was documented along the entire length of the organ of Corti, and the data were assembled into cochleograms. Student's *t* test was used to compare the number of missing outer hair cells between groups.

### Cochlear tissue collection for transcriptional analysis

To define site-specific changes in gene expression, we collected two types of cochlear samples: an organ of Corti sample and a non-organ of Corti sample. The organ of Corti sample contained sensory cells (inner and outer hair cells) and adjacent supporting cells (Deiters cells, pillar cells, Hensen cells, inner phalangeal cells and inner border cells). This cochlear sample was collected to determine sensory cell-related changes. The non-organ of Corti sample consisted of the cells in the lateral wall and the basilar membrane, and contained all of the cells in the stria vascularis and the spiral ligament, as well as mesothelial cells, the basement membrane, immune cells, Claudius cells and Boettcher cells. The dissection method used for collection of these tissues has been described in our previous studies.^39^ Briefly, at a defined time point before or after acoustic trauma (1, 4 or 7 days post noise exposure), the animals were killed by CO_2_ asphyxiation and were decapitated. The cochlea was quickly removed and placed in ice-cold Dulbecco's PBS (GIBCO, Life Technologies, Grand Island, NY, USA). The bony shell facing the middle ear cavity was quickly removed to expose the cochlear structure. Then, the tissue was placed in RNAlater solution to collect the organ of Corti tissue from the upper portion of the first cochlear turn. Then, the remaining tissue in the basilar membrane and the lateral wall tissues were collected. The isolated tissues were transferred to a small dish containing fresh RNAlater solution to wash out tissue debris from the surface of the samples. Then, the tissues were transferred to an RNase-free PCR tube and stored at −80 °C until the analysis of gene expression. The tissue from one cochlea was used to generate one organ of Corti sample and one lateral wall/basilar membrane sample.

### Quantitative reverse transcription-polymerase chain reaction (qRT-PCR)

qRT-PCR was performed to determine the transcriptional expression of five pro-inflammatory genes (Il6, Il1b, Tnf, Ccl2, and Ccl4), three MHC II-encoding genes (H2-Aa, H2-Eb1 and H2-M3) and two reference genes (Rpl13a and Hsp90ab). Total RNA was extracted from the collected tissues using an RNA extraction kit (RNeasy Plus Micro Kit; Qiagen GmbH, Hilden, Germany) and was reverse transcribed using a high capacity cDNA reverse transcription kit (Applied Biosystems, Foster City, CA, USA). The transcriptional expression levels were examined using pre-developed TaqMan gene expression primer/probe assays (Applied Biosystems). Pre-developed *Hsp90ab* and *Rpl13a* gene expression assays (Applied Biosystems) were used as endogenous controls. qRT-PCR was performed using a MyIQ two color Real-Time PCR detection system (Bio-Rad, Hercules, CA, USA).

To analyze changes in the expression of the target genes, we used a relative quantification method^40^ (Stankovic and Corfas, 2003). The expression level of a given gene was first normalized to the average level of the reference genes to generate the ΔCt value. The ΔΔCt was then calculated with the following formula: ΔCt (an experimental group)—ΔCt (a control group). The significance of the changes was analyzed using Student's *t* test or one-way ANOVA.

### Immunolabeling of immune proteins

Immunolabeling was performed to determine the protein expression of CD45, F4/80, MHC II and Tlr4 in immune cells beneath the basilar membrane. After the animals were killed, the cochleae were collected and fixed with 10% buffered formalin overnight at 4 °C. After fixation, the cochlea was dissected in PBS to collect the sensory epithelium under a dissection microscope. The tissues were treated with 0.5% Triton X-100 and 10% donkey or goat serum in PBS (pH 7.4) for 1 h at room temperature. Then, the tissues were incubated overnight at 4 °C with one primary antibody or two primary antibodies (for details regarding double-labeling of two proteins, see the Results section). The primary antibodies used and their dilutions were as follows: goat anti-CD45 polyclonal antibody (1 : 200, AF114, R&D Inc., Minneapolis, MN, USA); rat anti-F4/80 monoclonal antibody (1 : 150, ab6640, Abcam Inc., Cambridge, MA, USA); rabbit anti-TLR4 antibody (1 : 100, ab13556, Abcam Inc., Cambridge, MA, USA), and rat anti-MHC Class II monoclonal antibody (1 : 75, ab25333, Abcam Inc., USA).

After incubation with the primary antibodies, the tissues were rinsed with PBS (3 ×) and incubated in the dark with one secondary antibody or two secondary antibodies for 1 h at room temperature. The secondary antibodies (Life Technologies, Grand Island, NY, USA) and dilutions used were as follows: CD45 staining: Alexa Fluor 488 donkey anti-goat IgG, 1 : 200 in PBS; F4/80 and MHC Class II staining: Alexa Fluor 488 or 594 donkey anti-rat IgG, 1 : 100 in PBS; Tlr4: Alexa Fluor 488 donkey anti-rabbit IgG, 1 : 100 in PBS. After incubation, the samples were rinsed in PBS and mounted on slides in an antifade medium (Prolong Gold antifade reagent, Life Technologies, Carlsbad, CA, USA). Certain tissues were counterstained with propidium iodide (5 μg/ml in PBS) in the dark for 10 min at room temperature to demarcate nuclear morphology. To prevent false positive identifications, we examined the nonspecific staining of the secondary antibodies by omitting the primary antibody and found no clear fluorescence in the tissues. The specificity of the primary antibodies has been verified by a Western blot analysis in our previous studies.^7, 19^

### Fluorescence and confocal microscopy and immune cell quantification

The immunolabeled tissues were inspected using a fluorescence microscope (Leica Z6 APO Manual MacroFluo, Leica Microsystems Inc., Chicago, IL, USA) equipped with epifluorescence illumination to identify immune and sensory cells. The entire length of the basilar membrane was photographed, and the collected images were used for quantitative analyses of basilar membrane immune cells. The lesion sites were further examined via confocal microscopy (LSM510 multichannel laser scanning confocal imaging system, Zeiss, Thornwood, NY, USA) using a method that has been reported previously.^7, 19^

To quantify the protein expression of immune molecules, we identified cells that had stained positively for target proteins (see the Results section for details). Positive cells were defined by the presence of strong immunoreactivity relative to their neighboring cells. We counted the number of positive cells for each unit distance of the basilar membrane of each cochlea. Then, the numbers were averaged to generate a mean value for each group. Student's *t* test, one-way ANOVA or two-way ANOVA were performed to compare the group means.

### DQ-ovalbumin assay

To assess the macrophage functions of protein internalization, denaturation, reduction and proteolysis, we used ovalbumin conjugated with a self-quenching florescent dye. This probe exhibits bright green fluorescence upon proteolytic degradation. DQ-ovalbumin was purchased from Life Technology. Mice were sacrificed under a deep anesthesia. The cochleae were quickly collected and placed in 10 mM PBS at room temperature. Under a dissection microscope, the bony shell of the cochlea that faces the middle ear cavity was removed to expose the intra-cochlear structure. Then, the cochlea was incubated in the ovalbumin solution (50 *μ*g/ml in 10 mM PBS) for 1 h at 37 °C. After the staining, the cochlea was gently rinsed with 10 mM PBS (3 ×) and then was fixed with 10% buffered formalin. The cochlea was dissected and the sensory epithelium was collected. The collected tissue was mounted on a slide containing an antifade medium and examined under a fluorescence microscope. The stained samples were photographed with a confocal microscope (LSM510 multichannel laser scanning confocal imaging system, Zeiss, Thornwood, NY, USA). Positively stained cells beneath the basilar membrane in the middle and basal sections of the cochlea were counted for each unit distance. Then, the numbers were averaged to generate a mean value for each group. Two-way ANOVA were performed to compare the group means (noise factor × strain factor).

### Data analyses

All experimental comparisons were statistically analyzed using SigmaStat (Systat Software, Inc., San Jose, CA, USA) or GraphPad Prism (GraphPad Software, Inc., La Jolla, CA, USA). The data are presented as the mean±1 S.D. An α-level of 0.05 was selected for significance for all statistical tests.

## Abbreviations

Tlr4: Toll-like receptor 4
MHC II: major histocompatibility complex class II
ABR: auditory brainstem response
qRT-PCR: quantitative reverse transcription-polymerase chain reaction
PBS: phosphate-buffered saline
